# A comparison of first-attempt cannulation success of peripheral venous catheter systems with and without wings and injection ports in surgical patients—a randomized trial

**DOI:** 10.1186/s12871-022-01631-7

**Published:** 2022-03-31

**Authors:** Rudolf Mörgeli, Katrin Schmidt, Tim Neumann, Jochen Kruppa, Ulrich Föhring, Pascal Hofmann, Peter Rosenberger, Elke Falk, Willehad Boemke, Claudia Spies

**Affiliations:** 1 Department of Anesthesiology and Operative Intensive Care Medicine (CCM, CVK), Charité–Universitätsmedizin Berlin, corporate member of Freie Universität Berlin, Humboldt-Universität Zu Berlin, Berlin Institute of Health, Charitépl. 1, 10117 Berlin, Germany; 2 Department of Anesthesiology and Operative Intensive Care Medicine (CBF), Charité–Universitätsmedizin Berlin, corporate member of Freie Universität Berlin, Humboldt-Universität Zu Berlin, Berlin Institute of Health, Berlin, Germany; 3 Universitätsklinik Für Anästhesiologie Und Intensivmedizin Tübingen, Universitätsklinikum Tübingen, 72076 Tübingen, Germany

**Keywords:** Catheters, Catheterization, Peripheral, Cannulation, Operating room

## Abstract

**Background:**

A peripheral venous catheter (PVC) is the most widely used device for obtaining vascular access, allowing the administration of fluids and medication. Up to 25% of adult patients, and 50% of pediatric patients experience a first-attempt cannulation failure. In addition to patient and clinician characteristics, device features might affect the handling and success rates. The objective of the study was to compare the first-attempt cannulation success rate between PVCs with wings and a port access (Vasofix® Safety, B. Braun, abbreviated hereon in as VS) with those without (Introcan® Safety, B. Braun, abbreviated hereon in as IS) in an anesthesiological cohort.

**Methods:**

An open label, multi-center, randomized trial was performed. First-attempt cannulation success rates were examined, along with relevant patient, clinician, and device characteristics with univariate and multivariate analyses. Information on handling and adherence to use instructions was gathered, and available catheters were assessed for damage.

**Results:**

Two thousand three hundred four patients were included in the intention to treat analysis. First-attempt success rate was significantly higher with winged and ported catheters (VS) than with the non-winged, non-ported design (IS) (87.5% with VS vs. 78.2% with IS; *P*_Chi_ < .001). Operators rated the handling of VS as superior (rating of “good” or “very good: 86.1% VS vs. 20.8% IS, *P*_Chi_ < .001). Reinsertion of the needle into the catheter after partial withdrawal—prior or during the catheterization attempt—was associated with an increased risk of cannulation failure (7.909, CI 5.989–10.443, *P* < .001 and 23.023, CI 10.372–51.105, *P* < .001, respectively) and a twofold risk of catheter damage (OR 1.999, CI 1.347–2.967, *P* = .001).

**Conclusions:**

First-attempt cannulation success of peripheral, ported, winged catheters was higher compared to non-ported, non-winged devices. The handling of the winged and ported design was better rated by the clinicians. Needle reinsertions are related to an increase in rates of catheter damage and cannulation failure.

**Trial registration:**

ClinicalTrials.gov, Identifier: NCT02213965, Date: 12/08/2014.

**Supplementary Information:**

The online version contains supplementary material available at 10.1186/s12871-022-01631-7.

## Introduction

A peripheral venous catheter (PVC) is the most widely used device for obtaining continuous vascular access, allowing the administration of fluids and medication to a variety of patients. It is estimated that 30–80% of all hospitalized patients receive a PVC during their stay [1–4]. For procedural and safety reasons, vascular access in the surgical settings is virtually indispensable. Along with attachment of monitoring devices, the insertion of a PVC usually occurs immediately upon arrival in the induction or operating room, allowing for the administration of fluids, induction of anesthesia and management of potential complications.

Since establishing venous access occurs while the patient is awake, and usually precedes administration of any anesthetic agents, PVC placement can be an uncomfortable and painful procedure. Repeated and unsuccessful attempts to place a PVC can be stressful for both patient and clinician, causing localized pain and swelling, creating a portal of entry for micro-organisms, and forcing clinicians to seek alternative sites, where cannulation may be more difficult or dangerous [5–7]. Therefore, a successful PVC placement in the first attempt is always desirable. Reports indicate that up to a quarter of adult patients, and half of pediatric patients experience a first-attempt cannulation failure [8–10].

**Fig. 1:**
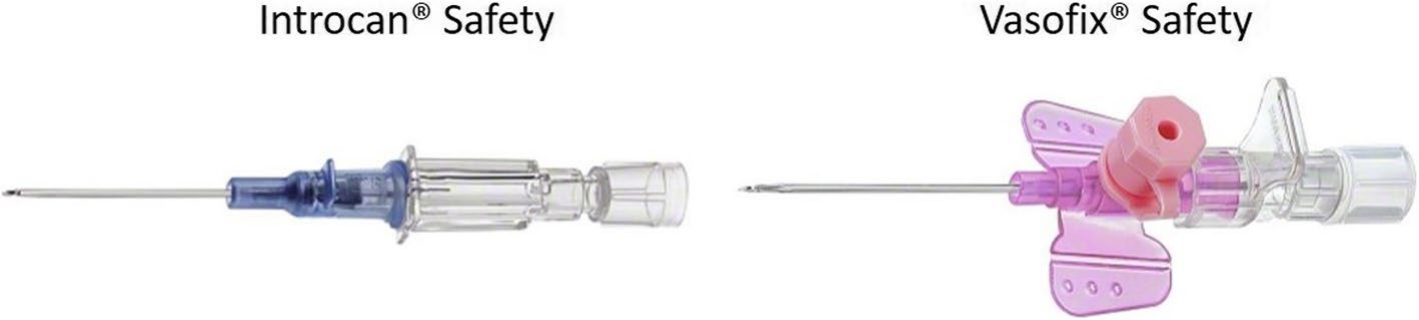
Introcan® Safety (IS) and Vasofix® Safety (VS), B. Braun Melsungen AG, Germany (images retrieved from https://www.bbraun.de/de/products/b0/vasofix-safety.html and https://www.bbraun.de/de/products/b/introcan-safety.html, adapted and printed with permission from B. Braun Melsungen)

Cannulation success rates can be influenced by the venous conditions and body-mass index (BMI) of the patient, as well as the level of experience of the healthcare provider [11], but also the type of PVC used may impact success rates. There are several types of PVCs available in the European market, and features such as size, bevel type, and the presence of notches, wings and ports might affect the handling of these devices [12]. Ported and winged catheters are widespread in German hospitals, but there is no evidence that they are superior to non-ported catheters, especially in relation to first-attempt cannulation success. The objective of this study is to compare the rate of first-attempt placement success between two PVC designs, namely a ported, winged catheter (Vasofix® Safety, B. Braun Melsungen AG, Germany, abbreviated hereon in as VS) and a non-ported, non-winged catheter (Introcan® Safety, B. Braun Melsungen AG, Germany, abbreviated hereon in as IS) (see Fig. 1).

## Methods

This open-label, multicenter study randomized patients to receive one of two distinct types of PVCs prior to elective surgery. This sponsor-initiated study compared two CE-marked, Class II medical devices in accordance with their intended purpose and use instructions [13, 14]. Data was collected between December 2014 and March 2016 by the anesthesiology departments of two German tertiary care university hospitals, namely the Charité – Universitätsmedizin Berlin with its three separate sites, Campus Charité Mitte (CCM), Campus Virchow Klinikum (CVK), and Campus Benjamin Franklin (CBF), and the Universitätsklinikum Tübingen. In accordance with inclusion and exclusion criteria (see Supplemental Fig. 1), adult and pediatric patients undergoing elective surgery were recruited by the anesthesiology staff during the routine pre-surgical assessment, and written informed consent was obtained from all participants or their proxies. Peripheral venous cannulation was required for surgery, so that patients were not subjected to any additional procedure or distress in the context of this investigation. All catheters were placed immediately prior to surgery, at the induction or operating room. The study was approved by the ethic committees of both participating universities (Berlin: EA2/104/14; Tübingen: 494/2014BO2), as well as by their respective data protection officers. The study was conducted in accordance with the Declaration of Helsinki and its amendments and was registered at ClinicalTrials.gov (NCT 02213965, 12/08/2014).

After providing informed consent, patients were randomly selected to receive a ported, winged fluorethylene propylene catheter (VS) or a non-ported, non-winged polyurethane catheter (IS). Study personnel prepared and conducted randomization via sealed envelopes, stratified by study site in a 1:1 ratio. General patient data was then collected, including age, gender, BMI, American Society of Anesthesiologists Physical Status (ASA-PS) score, as well as venous status (subjective assessment: good, moderate, or poor). Whenever possible, study personnel accompanied patients to the operating theater to ensure that venous cannulation took place with the designated catheter and that documentation was completed appropriately. The staff member placing the PVC (operator) completed a paper-based case report form (CRF), which included questions regarding the number and sites of cannulation attempts, subjective reasons for failure, adherence to use instructions (assessed by documenting needle reinsertion into the catheter, i.e. the practice of moving the needle stylet in the cannula prior to puncture or during the catheterization attempt; the manufacturer’s instructions for use (IFU) explicitly warn against this practice, and the variable was included as a marker for misuse), catheter handling information (e.g. 6-point Likert scale, blood spillage, use of ports), as well as their professional function and level of experience with venous catheterization. In case of a failed cannulation attempt, the catheter was collected and sent to the Bergische Universität Wuppertal, Faculty of Mechanical and Safety Engineering for a macro- and microscopic examination for signs of damage. Observation continued until patient discharge from the operating or recovery room, whereas any adverse events (e.g. catheter occlusion, displacement, or extravasation) were noted and the respective catheters collected. Used catheters were also collected following decannulation of ambulatory patients. No treatment changes occurred, all standard operating procedures remained in place throughout the investigation, and the attending physician had ultimate authority in all treatment decisions.

The primary outcome was successful cannulation at first attempt. Secondary outcomes included overall number of cannulation attempts, subjective reasons for cannulation failure, operators’ evaluation of catheter handling, frequency of needle reinsertion into the catheter, rate of PVC damage, and adverse events.

The sample size calculation, which was aimed for a power of 80%, an α of 0.05, and a 5% delta for a 75% first-attempt cannulation success for the control group (VS), yielded 1134 patients per trial arm (*n* = 2268). Additionally, in order to retain the prognostic balance provided by the randomization process [15], all data were analyzed according to the intention-to-treat (ITT) principle. Metric data was not normally distributed, so that data was reported as median and interquartile ranges and differences among groups were evaluated using the Wilcoxon-Mann–Whitney U test. Ordinal and nominal data were reported using frequencies and percentages, and groups were compared using Chi-Square Test, or Fisher’s Exact Test when small cell exceptions were present. Missing data was treated as such, with no replacement by estimates. Univariate and multiple logistic regression analyses was conducted to determine the most relevant factors for cannulation success at the first attempt, adjusting for possible confounders. The stepwise regression model was based on the available literature, which was supplemented by input from the stakeholders, who were familiar with the complaints and expectations from users in the field. The included variables depict patient (age, gender, BMI, cannulation site and venous condition) [16], operator (function and experience) [17], and product (catheter type and size) [17, 18] characteristics that might affect the cannulation success rates. Additionally, as part of an exploratory analysis, rate of needle reinsertion into catheters (any, before, or during the catheterization attempt) was added among operator variables as a misuse indicator, along with a detailed risk assessment for cannulation failure and catheter damage. Statistical analysis was performed with SPSS (Version 27.0, Armonk, NY: IBM Corp., USA).

## Results

Among the four study sites, 2304 patients were included in the ITT analysis, as shown in Supplemental Fig. 1 (Consort Diagram). Of these patients, 1133 received an IS catheter and 1171 received a VS catheter. Overall, there were no statistically relevant differences among patients in terms of age, gender, BMI, ASA score, venous conditions, or cannulation site. However, significant differences were observed in terms of catheter size (see Table 1). Patient characteristics among sites are provided in Supplemental Tables 1 and 2.

**Table 1:** All data shown as frequencies and percentages. *BMI* body-mass index, *ASA* American Society of Anesthesiologists. Missing data is treated as such

Patient Characteristics	N	All	Introcan	Vasofix	*P*
			**(*n* = 1134)**	**(*n* = 1175)**	
**Age (years)**	2304				.15
** 1 to 6**		34 (1.5%)	16 (1.4%)	18 (1.5%)	
** 7 to 12**		25 (1.1%)	13 (1.1%)	12 (1.0%)	
** 13 to 17**		43 (1.9%)	28 (2.5%)	15 (1.3%)	
** 18 to 44**		659 (28.6%)	311 (27.4%)	348 (29.7%)	
** 45 to 64**		840 (36.5%)	432 (38.1%)	408 (34.8%)	
** ≥ 65**		703 (30.5%)	333 (29.4%)	370 (31.6%)	
**Gender**	2279				.43
** Female**		1097 (48.1%)	532 (47.3%)	565 (49.0%)	
**BMI (adults only)**	2175				.62
** < 18.5 kg/m**^**2**^		51 (2.3%)	25 (2.4%)	26 (2.3%)	
** 18.5 to 24.9 kg/m**^**2**^		951 (43.7%)	471 (44.4%)	480 (43.1%)	
** 25 to 29.9 kg/m**^**2**^		719 (33.1%)	337 (31.7%)	382 (34.3%)	
** ≥ 30 kg/m**^**2**^		454 (20.9%)	229 (21.6%)	225 (20.2%)	
**ASA Score**	2242				.65
** 1**		600 (26.8%)	303 (27.7%)	297 (25.8%)	
** 2**		1287 (57.4%)	623 (57.0%)	664 (57.8%)	
** 3**		349 (15.6%)	165 (15.1%)	184 (16.0%)	
** 4**		6 (0.3%)	2 (0.2%)	4 (0.3%)	
**Venous Status**	2277				.58
** Good**		1583 (69.5%)	778 (69.4%)	805 (69.6%)	
** Moderate**		568 (24.9%)	286 (25.5%)	282 (24.4%)	
** Poor**		126 (5.5%)	57 (5.1%)	69 (6.0%)	
**Catheter Size**	2295				.045
** ≤ 16 G**		86 (3.7%)	32 (2.8%)	54 (4.6%)	
** 18 G**		1185 (51.6%)	587 (52.1%)	598 (50.5%)	
** 20 G**		951 (41.4%)	478 (42.5%)	473 (40.5%)	
** ≥ 22 G**		73 (3.2%)	29 (2.6%)	44 (3.8%)	
**Site of Cannulation**	2290				.44
** Back of hand**		1814 (79.2%)	905 (80.3%)	909 (78.2%)	
** Forearm**		339 (14.8%)	153 (13.6%)	186 (16.0%)	
** Antecubital fossa**		110 (4.8%)	55 (4.9%)	55 (4.7%)	
** Other**		27 (1.2%)	14 (1.2%)	13 (1.1%)	
**Operator Function**	2275				.88
** Physician**		834 (36.7%)	416 (37.1%)	418 (36.2%)	
** Registered Nurse**		1250 (54.9%)	610 (54.4%)	640 (55.5%)	
** Other Specialist**		191 (8.4%)	95 (8.5%)	96 (8.3%)	
**Operator Experience**	2166				.31
** < 3 years**		486 (22.4%)	248 (23.4%)	238 (21.5%)	
** > 3 years**		1680 (77.6%)	813 (76.6%)	867 (78.5%)

**Table 2:** All data shown as frequencies and percentages. *ITT* intention to treat, *BMI* body-mass index. Missing data is treated as such

Successful Cannulation	Introcan (IS)			Vasofix (VS)			*P*
	n total	n successful	%	n total	n successful	%	
**Total (ITT)**	1133	886	78.2%	1171	1025	87.5%	< .001
**Age (years)**							
** 1 to 6**	16	12	75.0%	18	10	55.6%	.24
** 7 to 12**	13	12	92.3%	12	9	75.0%	.32
** 13 to 17**	28	23	82.1%	15	13	86.7%	.70
** 18 to 44**	311	257	82.6%	348	305	87.6%	.07
** 45 to 64**	432	330	76.4%	408	365	89.5%	< .001
** ≥ 65**	333	252	75.7%	370	323	87.3%	< .001
**Gender**							
** Female**	532	418	78.6%	565	474	83.9%	.02
** Male**	593	463	78.1%	589	538	91.3%	< .001
**BMI (adults only)**							
** < 18.5 kg/m**^**2**^	25	21	84.0%	26	23	88.5%	.70
** 18.5 to 24.9 kg/m**^**2**^	471	365	77.5%	480	427	89.0%	< .001
** 25 to 29.9 kg/m**^**2**^	337	266	78.9%	382	334	87.4%	.002
** ≥ 30 kg/m**^**2**^	229	175	76.4%	225	197	87.6%	.002
**Venous Condition**							
** Good**	778	657	84.4%	805	745	92.5%	< .001
** Moderate**	286	198	69.2%	282	234	83.0%	< .001
** Poor**	57	23	40.4%	69	35	50.7%	.25
**Site of Cannulation**							
** Back of hand**	905	714	78.9%	909	816	89.8%	< .001
** Forearm**	153	121	79.1%	186	155	83.3%	.38
** Antecubital fossa**	55	40	72.7%	55	41	74.5%	.83
**Operator Function**							
** Physicians**	416	312	75.0%	418	364	87.1%	< .001
** Nurses**	610	483	79.2%	640	560	87.5%	< .001
** Others**	95	80	84.2%	96	88	91.7%	.11
**Operator Experience**							
** > 3 years**	813	655	80.6%	867	762	87.9%	< .001
** < 3 years**	248	175	70.6%	238	207	87.0%	< .001
**Catheter Size**							
** ≤ 16 G**	32	28	87.5%	54	52	96.3%	.19
** 18 G**	587	472	80.4%	598	546	91.3%	< .001
** 20 G**	478	356	74.5%	473	399	84.4%	< .001
** ≥ 22 G**	29	24	82.8%	44	26	59.1%	.03

Overall, the first-attempt cannulation success was achieved in 82.9% of cases and was higher with VS (87.5%) than with IS (78.2%) (*P* < 0.001). This significance was confirmed in a per protocol group analysis (87.5% vs. 80.5% success rate; *n* = 2267, *P* < 0.001), as well as in a sub-analysis including only patients ≥ 18 years of age (88.2% vs. 78.0% success rate; *n* = 2202, *P* < 0.001). Relevant factors influencing first-attempt success rates are shown in Table 2. While the success rates with VS were significantly higher in patients with at least 45 years of age, differences ceased to be significant for those aged between 18 and 44, and observed success rates did not differ significantly for patients below the age of 18. There were no differences in success rates among patients with a BMI < 18.5 kg/m^2^, although VS was superior in all other BMI categories. VS was also superior in good and moderate vein conditions, while the success rate was not significantly different in patients with poor venous status. The majority of venous catheters were placed on the back of the hand (78.7%), where success rates with VS were significantly higher than at other cannulation sites. Here, a success rate of 89.8% was achieved with VS, compared to 78.9% with IS. No statistical differences were detected between success rates for cannulas placed in the forearm or antecubital fossa. Compared to IS, first-attempt success rates were significantly higher with VS for nurses and physicians alike (*P* < 0.001), although this difference could not be observed among other groups (i.e. medical students, interns). There were differences among centers regarding operator function. While physicians performed the majority of cannulations in CBF and UCT (60.7% and 54.6%, respectively), nurses performed the majority of cannulations in CCM and CVK (83.7% and 61.4%, respectively). 72.9% of operators had over 3 years of work experience, placing an average of 17 (± 9) catheters per week. Here, nurses placed significantly higher number of catheters per week in comparison to physicians (*P* < 0.001), and only 7% reported having less than 3 years of experience, compared to 50% of physicians. The multivariate analysis (*n* = 1157) identified venous conditions, BMI, operator function and experience, adherence to IFU, catheter size and catheter type as the most significant determinants of cannulation success at the first attempt (see Table 3). In a regression model without misuse markers (*n* = 1956, Supplemental Table 3), operator function ceased to be significant.

**Table 3:** Multivariate analysis; *VS* Vasofix® Safety, *IS* Introcan® Safety; adult participants only; All data analyzed as categorical variables

Multivariate Analysis (Risk of Cannulation Failure)	n	*P*	Odds Ratio	95% Confidence Interval
Patient Variables				
Age	1157	.74	0.998	0.988 … 1.008
Gender, female (0) / male	619/538	.18	1.275	0.893 … 1.820
Body Mass Index				
< 18.5 kg/m^2^	29	.54	0.691	0.210 … 2.279
18.5 to 24.9 kg/m^2^	514	.07		
25 to 29.9 kg/m^2^	391	.92	0.980	0.669 … 1.434
≥ 30 kg/m^2^	223	.01	0.557	0.351 … 0.885
Venous Condition				
Good (0)	787	< .001		
Moderate	295	< .001	2.837	1.945 … 4.138
Poor	75	< .001	10.780	5.665 … 20.512
Site of Cannulation				
Back of hand (0)	887	.43		
Forearm	184	.37	1.230	0.781 … 1.938
Antecubital fossa	71	.23	1.494	0.776 … 2.876
Other	15	.32	1.843	0.552 … 6.152
Operator Variables				
Operator Function				
Nurses (0)	701	.02		
Physicians	397	.005	1.756	1.188 … 2.597
Others	59	.98	0.989	0.445 … 2.199
Experience				
> 3 years (0) / < 3 years	886/271	.21	1.311	0.858 … 2.003
Needle Reinsertion				
Pre-puncture, No (0) / Yes	891/266	< .001	7.663	5.353 … 10.970
Post-puncture, No (0) / Yes	1106/51	< .001	15.401	6.271 … 37.824
Product Variables				
Catheter type, VS (0) / IS	587/570	< .001	2.996	2.134 … 4.207
Catheter size				
≤ 16 G	32	.03	0.208	0.050 … 0.867
18 G (0)	652	.003		
20 G	468	.03	1.478	1.044 … 2.093
≥ 22 G	5	.046	10.895	1.049 … 113.181

The most commonly reported reasons for cannulation failure were poor vein status (48.8% of respondents, *n* = 313), poor handling (27.8% or respondents, *n* = 127; 90.8% IS vs. 9.2% VS, *P* < 0.001), and blunt cannulas (21.4% of respondents, *n* = 67; 76.1% IS vs. 23.9% VS, *P* = 0.73). Handling of VS was rated by operators as “good” or “very good” in 86.1% of cases, while only 20.8% rated IS as such (*P* < 0.001) (see Table 4). Accordingly, VS operators said that the wings and ports influenced catheter placement in 25.7% of cases. The port of the VS was used in 65.0% of cases for flushing, and/or drug administration.

**Table 4:** Handling characteristics. Subjective assessment by corresponding operators

**Handling Characteristics**	**Total**			**Introcan**			**Vasofix**			*Pchi*
	N (all)	N	Perc	N (all)	N	Perc	N (all)	N	Perc	
**Was backflow clearly/quickly visible?**	2293									< .001
** Yes**		2150	93.8%	1126	1025	91.0%	1167	1125	96.4%	
** No**		143	6.2%		101	9.0%		42	3.6%	
**Was there blood spillage during placement?**	2275									< .001
** Yes**		285	12.5%	1113	185	16.6%	1162	100	8.6%	
** No**		1990	87.5%		928	83.4%		1062	91.4%	
**Dressing status**	2216									< .001
** Clean, dry**		2131	96.2%	1068	1002	93.8%	1148	1129	98.3%	
** Soiled by blood**		33	1.5%		26	2.4%		7	0.6%	
** Soiled by other fluids**		44	2.0%		34	3.2%		10	0.9%	
** Soiled by blood and other fluids**		8	0.4%		6	0.6%		2	0.2%	
**Did the port or wings influence catheterization?**										
** No**							1138	846	74.3%	-
** Yes, the port**								71	6.2%	
** Yes, the wings**								81	7.1%	
** Yes, the port and wings**								140	12.3%	
**Overall Assessment**	2302									< .001
** Very good**		572	24.8%	1133	41	3.6%	1169	531	45.4%	
** Good**		671	29.1%		195	17.2%		476	40.7%	
** Satisfactory**		404	17.5%		303	26.7%		101	8.6%	
** Sufficient**		312	13.6%		268	23.7%		44	3.8%	
** Poor**		240	10.4%		228	20.1%		12	1.0%	
** Unsatisfactory**		103	4.5%		98	8.6%		5	0.4%	

Data on misuse parameters was limited with a 58.9% response rate. Partial needle withdrawal and reinsertion into the cannula prior to the catheterization attempt (to ensure mobility of the needle stylet) was reported in 318 of 1355 documented cases (23.5%), while the same maneuver during the catheterization attempt (i.e. partial needle withdrawal to test whether a flashback in the cannula occurs, followed by needle reinsertion and a further catheterization attempt) was reported in 61 of 1348 cases (4.5%). These maneuvers were associated with an 8- and 23-fold risk of cannulation failure, respectively (see Table 5; a detailed analysis by catheter is available in Supplemental Table 4). Of the 461 catheters returned for laboratory evaluation, 32.5% displayed signs of damage, and needle reinsertion prior or during the catheterization attempt were associated with a twofold increase in the risk of catheter damage (see Table 5; a detailed analysis by catheter is available in Supplemental Table 5).

**Table 5:** Catheter misuse in relation to risk of cannulation failure and catheter damage. Catheter misuse refers to needle movement, the practice of moving the needle in the cannula prior to puncture or during the catheterization attempt (the manufacturer’s use instructions explicitly warn against such maneuvers). Catheter damage refers to abnormalities in catheter structure, macroscopic or microscopic, such as cuts, tears, compressions or loss of material

**Needle Reinsertion (Misuse)**	**N**		**Odds Ratio**	**95% Confidence Interval**	***P***
Risk of Cannulation Failure		**Failure Rate**			
Needle Reinsertion	1358				
None (proper use)	1019 (75.0%)	162 (15.9%)			
Yes (misuse)	339 (25.0%)	219 (64.3%)	9.531	7.214 … 12.592	< .001
Prior to Catheterization Attempt	1355				
None (proper use)	1037 (76.5%)	181 (13.4%)			
Yes (misuse)	318 (23.5%)	199 (62.6%)	7.909	5.989 … 10.443	< .001
During Catheterization Attempt	1348				
None (proper use)	1287 (95.5%)	323 (25.1%)			
Yes (misuse)	61 (4.5%)	54 (88.5%)	23.023	10.372 … 51.105	< .001
Risk of Catheter Damage		**Damage Rate**			
Needle Reinsertion	461				
None (proper use)	253 (54.9%)	65 (25.7%)			
Yes (misuse)	208 (45.1%)	85 (40.9%)	1.999	1.347 … 2.967	.001
Prior to Catheterization Attempt	461				
None (proper use)	270 (58.6%)	73 (27.0%)			
Yes (misuse)	191 (41.4%)	77 (40.3%)	1.823	1.228 … 2.705	.003
During Catheterization Attempt	459				
None (proper use)	412 (89.8%)	127 (30.8%)			
Yes (misuse)	47 (10.2%)	23 (48.9%)	2.151	1.170 … 3.954	.01

Following an unsuccessful first attempt, two further attempts were usually required to secure venous access (75.9%; median 3, IQR [3, 3]), regardless of the type of cannula used. Overall, adverse events took place in 77 (3.3%) cases, whereas 65 of these events involved the IS cannula (*P* < 0.001). No serious adverse events were observed and only one needle stick injury took place during the trial (with a VS PVC), which took place prior to patient (or blood) contact.

## Discussion

In general, success rates were significantly higher with the ported, winged catheter VS than with the non-ported, non-winged IS. With 83% in the ITT and 84% in the per protocol (PP) populations, the success rates observed were higher than the 73–74% success rates reported in emergency departments [9, 11], but failed to reach the desired 90% threshold [19]. The results are comparable to recent studies including surgical patients, which report a 79–85% first-attempt success rate [12].

Key factors affecting first-attempt success rates of peripheral cannulation were described by Carr et al. as patient, operator, and product variables [20]. Patient factors included age, BMI, and venous status, while operator characteristics included function and experience. Product variables included catheter size, but this study could demonstrate that handling and differences in product design can also affect success rates. For the surgical cohort, the most significant factors in the multifactorial analysis were venous status, catheter type, higher BMI, and operator experience. Additionally, adherence to IFU (i.e. no needle reinsertion) was shown to be a significant factor, although the analysis encompassed only a subpopulation of the cohort. The operator-reported reasons for cannulation failure were most often poor venous status, poor handling of the PVC (primarily IS), and blunt needles.

Overall, the VS design was shown to be more favorable. This PVC design, which has wings and an injection port, appears to facilitate catheter placement and fixation, as indicated by the consistently higher successful cannulation rates, improved backflash visibility, as well as lower rates of spillage and soiled dressings. The majority of operators (86.1%) rated the VS PVC as “good” or “very good”, while IS received such ratings from only 20% of operators. The injection port also appears to be well accepted, being utilized during the surgical procedure for flushing and/or drug administration in approximately 2/3 of cases, despite the availability of 3-way stopcocks. It must be noted, however, that such ports have been associated with a greater risk of infection, likely due to the inability to properly decontaminate the port area [21].

Interestingly, the superiority of the VS PVC was diminished with younger age and lower BMI. The slimmer and smaller IS design seems to be more suitable for children and patients with BMI < 18.5 kg/m^2^, although these differences remained statistically insignificant, likely due to the smaller size of these subgroups. Nevertheless, IS PVCs were associated with 65 of the 77 (84.4%) catheter-related adverse events observed in this investigation. These included primarily extravasate infusions, dislocations, and occlusions, factors that are rather related to care and maintenance of the PVCs, and not the placement procedure itself.

The most commonly chosen site for cannulation in this surgical collective was the backside of the hand, which usually allows ease of access for anesthesiologists throughout the surgical procedure. Furthermore, should a cannulation failure occur, proximal alternative sites remain available. This remains the primary cannulation site despite evidence that placement in the forearm reduces catheter-associated complication rates [22].

Physicians and nurses achieved similar success rates. Nurse involvement increased when moderate or poor vein conditions were reported, whereas nurses were also significantly more experienced and placed a higher number of catheters per week in comparison to physicians, suggesting an advantage in terms of routine and experience. More experienced physicians are underrepresented, likely because they frequently supervise longer and more complex operations (often one per day), whereas less experienced colleagues usually accompany several short and simple procedures during a workday. When taking experience into consideration, there were no differences in success (or misuse) between physicians and nurses. Interestingly, the best success rates were achieved by students and interns, despite no differences in the subjective assessment of the patients’ venous status. This is possibly due to less time pressure and a selected patient group, but it must be noted that in the larger, more representative logistic regression (Supplemental Table 3), no significant differences were observed.

The PVCs were not always used as intended. The pervasive custom of moving the needle inside the plastic cannula prior to puncture took place in nearly a quarter of cases, being associated with an increase in the risk of failure from 16 to 64%. Likewise, partially withdrawing the needle through the cannula after a puncture, then reinserting it in a rescue attempt to redirect the cannula was seen in nearly 5% of cases, with an 88.5% likelihood of failure. The IFU specifically warn users not to reinsert needles through the cannula, and there are reports of intravenous catheter fractures and embolisms [23]. In this investigation, such maneuvers were related to a twofold increase in the rate of catheter damage in the laboratory analysis. While VS showed higher rates of damage, with or without needle reinsertion, IS appears to be more susceptible to damage by misuse, possibly due to the polyurethane material (see Supplemental Table 5). This information must be emphasized in training sessions in an effort to reduce such practices. It is also worthy of note that 25.7% of PVCs that were used properly still showed signs of damage.

It is important to note that an increasing number of tools have been developed for predicting difficult venous access, both for pediatric [24–26] and adult patients [19, 27–29]. Additionally, there has been a marked increase in the availability and acceptance of ultrasound-guided assessment and cannulation [30, 31], sparking the formation of vascular access teams [32]. The European Society of Anesthesiologist published guidelines recommending the early identification of difficult venous access through validated tools, as well as the use of ultrasound [33]. The implementation of such tools has the potential to improve patient care by adapting clinical processes and allowing an appropriate allocation of personnel and materials, as well as provide an objective basis for the comparison in clinical studies.

### Limitations

Several limitations must be taken into account. Although an open-label investigation was unavoidable, there was an attempt to minimize bias through implementation of a randomization process and the use of CRFs. Nevertheless, an observation bias cannot be ruled out, and nor can a performance bias (involvement of more experienced colleagues by particularly difficult venous status). Although patients were accompanied by study personnel whenever possible, self-reported data from unsupervised operators may be an additional source of bias. The venous status of patients was subjective and may vary considerably, depending on the operator’s experience. Although the adult version of the Difficult Intravenous Access (DIVA) scale [27] was not available at the beginning of this trial, the lack of an objective scoring system is a clear limitation of this study. No identifiable information was collected from the operators, and no data is available regarding the number of operators involved nor the frequency of their participation. No follow-up attempt was made after discharge from the recovery room, and the short duration of observation may have affected the reported rate of complications. There might be additional factors affecting cannulation success that were not considered in this study, particularly in the multivariate analyses. Although both products were newly introduced, it is important to note that winged and ported catheters are far more common in Germany, so that operators were likely to be more familiar with this design type. Additionally, due to the high number of missing values, data on needle reinsertion and damage must be regarded with caution. Data generalizability is limited, as the study was conducted exclusively in a university setting.

## Conclusions

First-attempt cannulation success was more likely with ported, winged VS catheters compared to non-ported, non-winged IS devices. The larger VS design allowed for improved handling and fixation, although this superiority could not be observed in younger patients or those with low BMI. The overall rate of successful puncture at first attempt in the operating room was 83%. Among clinicians, nurses were more experienced and placed the majority of catheters. The backside of the hand was the preferred site, and the most commonly reported reasons for failure were poor venous status and poor handling of the catheter. This trial identified the reinsertion of the needle stylet into the cannula, prior or during the cannulation attempt, as novel clinician-related markers that were associated with higher incidences of catheter damage and cannulation failure. This information must be highlighted in training sessions to limit these dangerous practices and improve patient safety. Standardized tools for the identification of difficult venous access are increasingly common, and the implementation of ultrasound techniques and dedicated vascular access teams have the potential to improve care and reduce catheter-related complications.

## Supplementary Information

**Additional file 1:** **Supplemental Figure 1.** Consort diagram, flowchart; *For one study site (Universitätsklinik Tübingen), only information about completed case report forms are available; # Other reasons include screening failure, urgent operations, and missing documentation; ## Other reasons include missing/incomplete data and drop-out participants.

**Additional file 2:** **Supplemental Table 1.** Patient characteristics by center. All data shown as frequencies and percentages. *ASA * American Society of Anesthesiologists, *CBF* Campus Benjamin Franklin, *CVK* Campus Virchow Klinikum, *CCM* Campus Charité Mitte; *UCT* Universitätsklinikum Tübingen; P-values represent Chi-Square Test, or Fisher’s Exact Test when small cell exceptions were present; missing data is treated as such.

**Additional file 3:** **Supplemental Table 2.** Patient characteristics by center (continued). All data shown as frequencies and percentages. *CBF* Campus Benjamin Franklin, *CVK* Campus Virchow Klinikum, *CCM* Campus Charité Mitte, *UCT* Universitätsklinikum Tübingen. Missing data is treated as such. Vein status is a subjective variable assigned by the corresponding operator; P-values represent Chi-Square Test, or Fisher’s Exact Test when small cell exceptions were present; missing data is treated as such.

**Additional file 4:** **Supplemental Table 3.** Multivariate analysis without misuse markers; VS: Vasofix® Safety, IS: Introcan® Safety; adult participants only; All data analyzed as categorical variables.

**Additional file 5:** **Supplemental Table 4.** Catheter misuse in relation to risk of cannulation failure, detailed by type of catheter. Catheter misuse refers to needle movement, the practice of moving the needle in the cannula prior to puncture or during the catheterization attempt (the manufacturer’s use instructions explicitly warn against such maneuvers).

**Additional file 6:** **Supplemental Table 5.** Catheter misuse in relation to risk of catheter damage, detailed by type of catheter. Catheter misuse refers to needle movement, the practice of moving the needle in the cannula prior to puncture or during the catheterization attempt (the manufacturer’s use instructions explicitly warn against such maneuvers). Catheter damage refers to abnormalities in catheter structure, macroscopic or microscopic, such as cuts, tears, compressions or loss of material.

## Data Availability

The datasets analyzed during the current study are not publicly available due to constrains imposed in the consent forms, but an anonymized version is available from the corresponding author on reasonable request.

## Abbreviations

PVC: Peripheral venous catheter
BMI: Body-mass index
VS: Vasofix® Safety
IS: Introcan® Safety
CBF: Charité Campus Benjamin Franklin
CVK: Charité Campus Virchow Klinikum
CCM: Charité Campus Charité Mitte
UCT: Universitätsklinikum Tübingen
CRF: Case report form
ITT: Intention-to-treat
PP: Per protocol
ASA: American Society of Anesthesiologists
OR: Odds Ratio
CI: Confidence Interval
