# Supplementary material for: A comparison of first-attempt cannulation success of peripheral venous catheter systems with and without wings and injection ports in surgical patients—a randomized trial
Source: BMC Anesthesiol. 2022 Mar 31;22:88. doi: 10.1186/s12871-022-01631-7 (PMC8969381; doi:10.1186/s12871-022-01631-7)
Supplement: Supplementary file 1 — Additional file 1: Supplemental Figure 1. Consort diagram, flowchart; *For one study site (Universitätsklinik Tübingen), only information about completed case report forms are available; # Other reasons include screening failure, urgent operations, and missing documentation; ## Other reasons include missing/incomplete data and drop-out participants. [file 12871_2022_1631_MOESM1_ESM.docx]

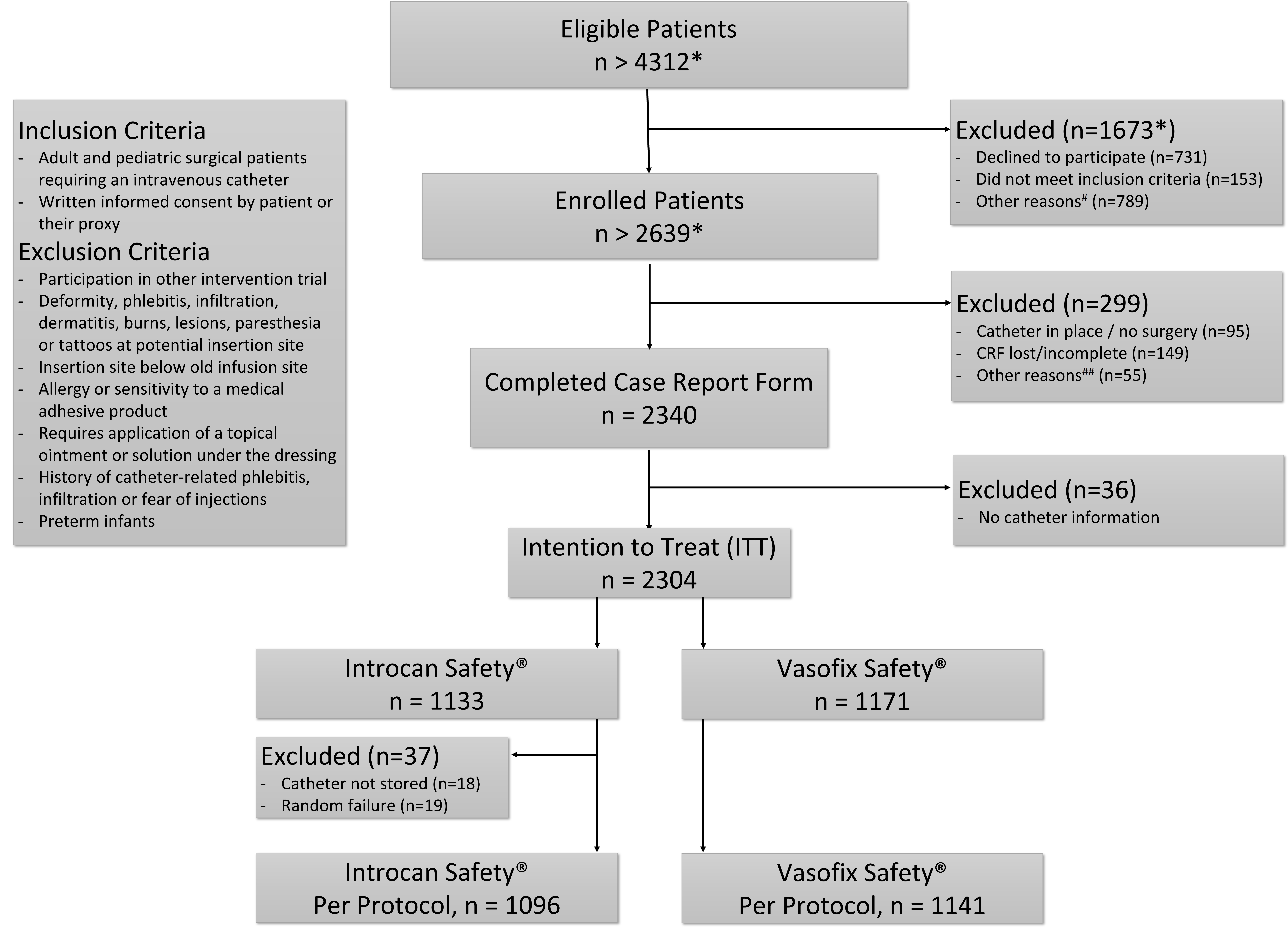


Supplemental Figure 1: Consort diagram, flowchart; *For one study site (Universitätsklinik Tübingen), only information about completed case report forms are available; # Other reasons include screening failure, urgent operations, and missing documentation; ## Other reasons include missing/incomplete data and drop-out participants.
