# Supplementary material for: A comparison of first-attempt cannulation success of peripheral venous catheter systems with and without wings and injection ports in surgical patients—a randomized trial
Source: BMC Anesthesiol. 2022 Mar 31;22:88. doi: 10.1186/s12871-022-01631-7 (PMC8969381; doi:10.1186/s12871-022-01631-7)

Supplemental Table 1: Patient characteristics by center. All data shown as frequencies and percentages. ASA: American Society of Anesthesiologists; CBF: Campus Benjamin Franklin; CVK: Campus Virchow Klinikum; CCM: Campus Charité Mitte; UCT: Universitätsklinikum Tübingen; *P*-values represent Chi-Square Test, or Fisher’s Exact Test when small cell exceptions were present; missing data is treated as such.


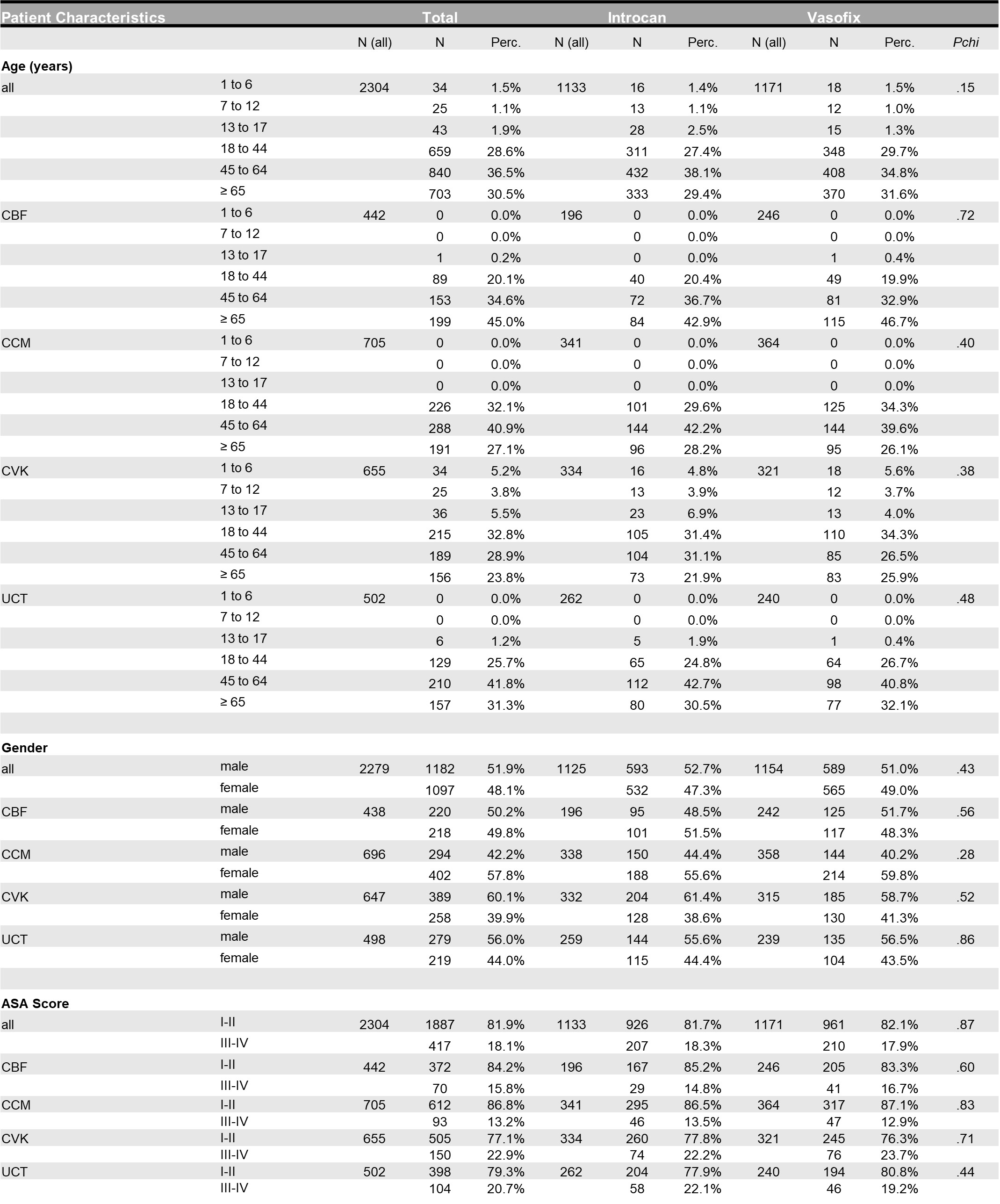

Supplement: Supplementary file 2 — Additional file 2: Supplemental Table 1. Patient characteristics by center. All data shown as frequencies and percentages. ASA American Society of Anesthesiologists, CBF Campus Benjamin Franklin, CVK Campus Virchow Klinikum, CCM Campus Charité Mitte; UCT Universitätsklinikum Tübingen; P-values represent Chi-Square Test, or Fisher’s Exact Test when small cell exceptions were present; missing data is treated as such. [file 12871_2022_1631_MOESM2_ESM.docx]
