# Supplementary material for: A comparison of first-attempt cannulation success of peripheral venous catheter systems with and without wings and injection ports in surgical patients—a randomized trial
Source: BMC Anesthesiol. 2022 Mar 31;22:88. doi: 10.1186/s12871-022-01631-7 (PMC8969381; doi:10.1186/s12871-022-01631-7)
Supplement: Supplementary file 3 — Additional file 3: Supplemental Table 2. Patient characteristics by center (continued). All data shown as frequencies and percentages. CBF Campus Benjamin Franklin, CVK Campus Virchow Klinikum, CCM Campus Charité Mitte, UCT Universitätsklinikum Tübingen. Missing data is treated as such. Vein status is a subjective variable assigned by the corresponding operator; P-values represent Chi-Square Test, or Fisher’s Exact Test when small cell exceptions were present; missing data is treated as such. [file 12871_2022_1631_MOESM3_ESM.docx]

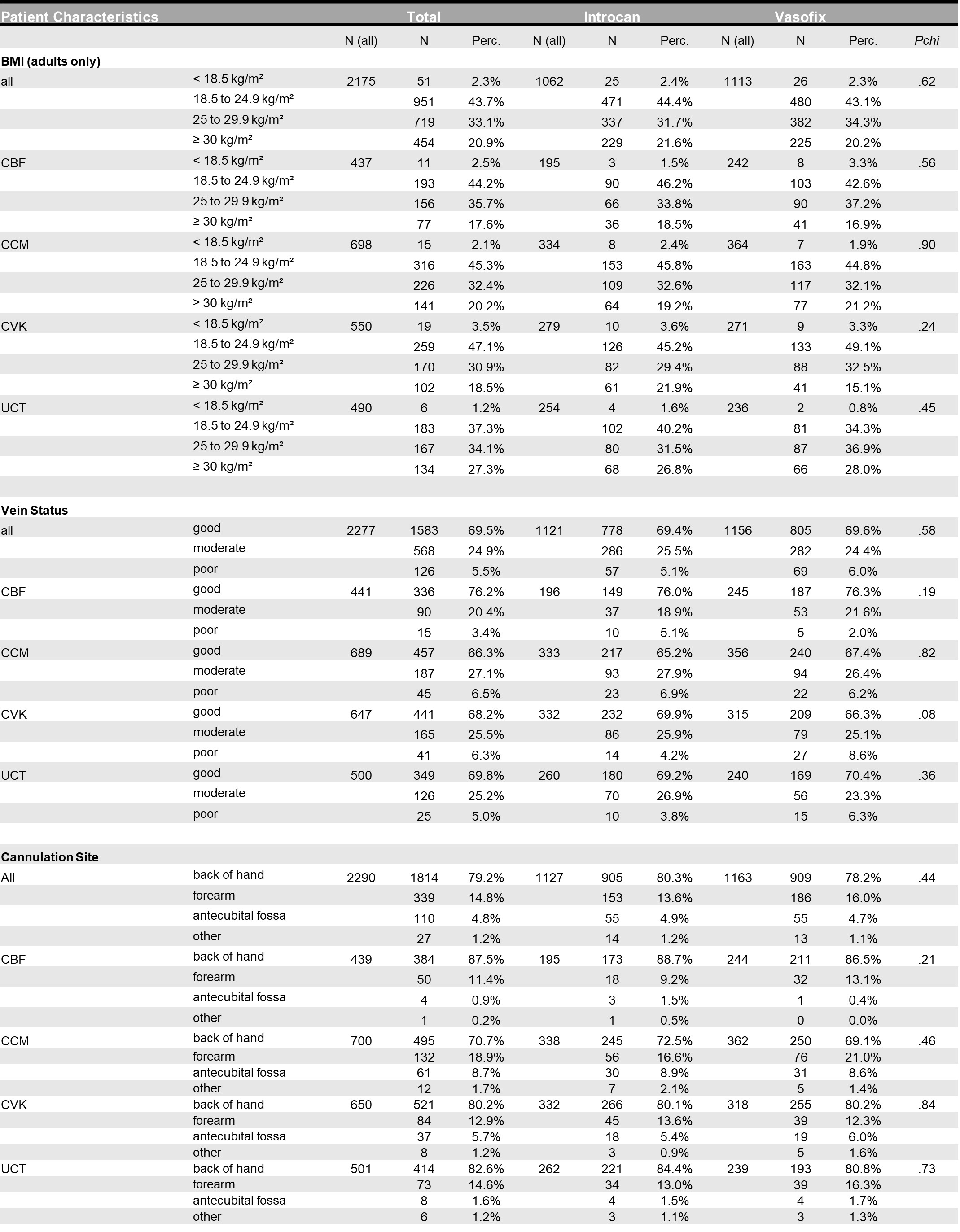


Supplemental Table 2: Patient characteristics by center (continued). All data shown as frequencies and percentages. CBF: Campus Benjamin Franklin, CVK: Campus Virchow Klinikum, CCM: Campus Charité Mitte, UCT: Universitätsklinikum Tübingen. Missing data is treated as such. Vein status is a subjective variable assigned by the corresponding operator; *P*-values represent Chi-Square Test, or Fisher’s Exact Test when small cell exceptions were present; missing data is treated as such.
