# Supplementary material for: A comparison of first-attempt cannulation success of peripheral venous catheter systems with and without wings and injection ports in surgical patients—a randomized trial
Source: BMC Anesthesiol. 2022 Mar 31;22:88. doi: 10.1186/s12871-022-01631-7 (PMC8969381; doi:10.1186/s12871-022-01631-7)
Supplement: Supplementary file 4 — Additional file 4: Supplemental Table 3. Multivariate analysis without misuse markers; VS: Vasofix® Safety, IS: Introcan® Safety; adult participants only; All data analyzed as categorical variables. [file 12871_2022_1631_MOESM4_ESM.docx]

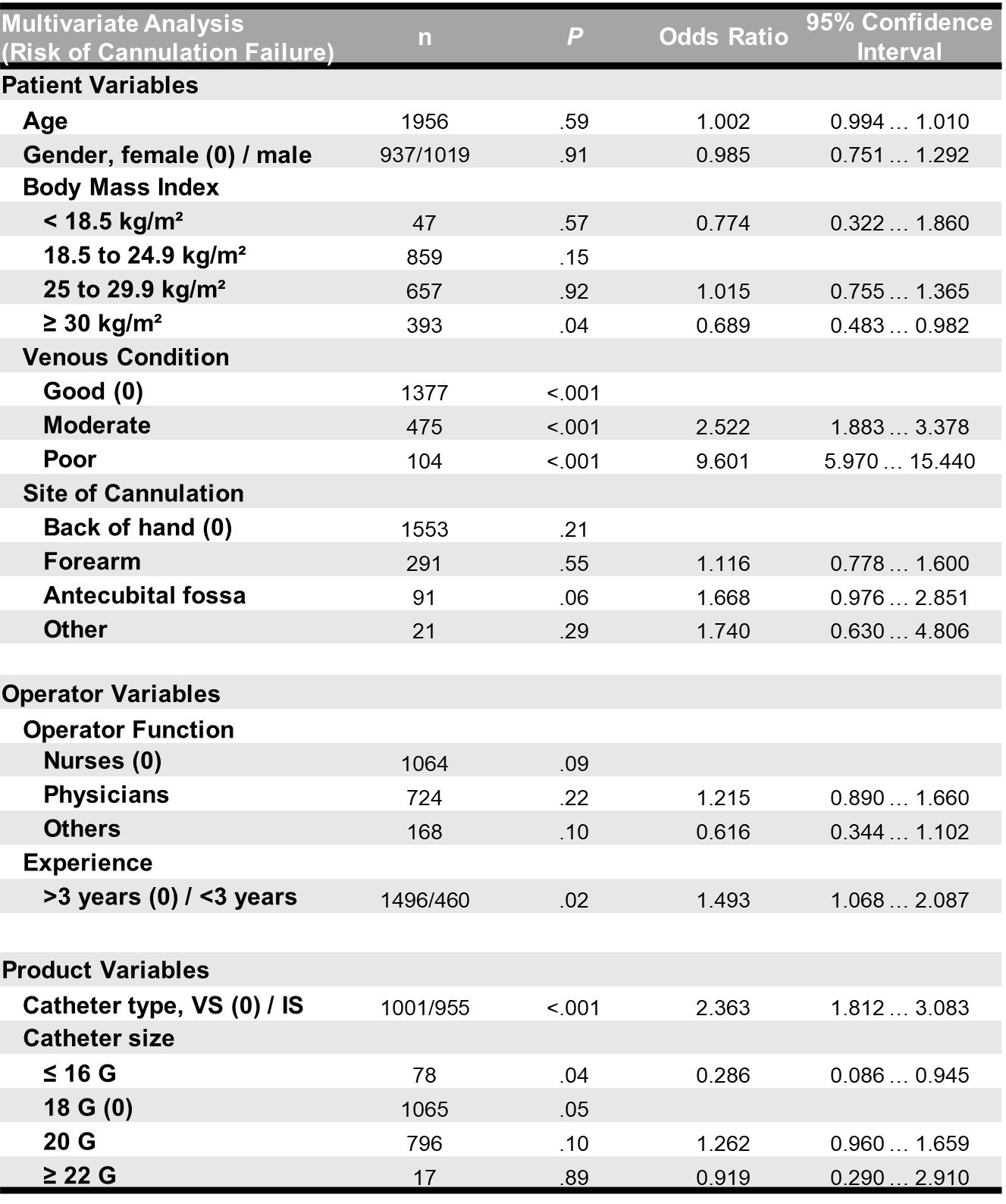


Supplemental Table 3: Multivariate analysis without misuse markers; VS: Vasofix® Safety, IS: Introcan® Safety; adult participants only; All data analyzed as categorical variables.
