# Supplementary material for: A comparison of first-attempt cannulation success of peripheral venous catheter systems with and without wings and injection ports in surgical patients—a randomized trial
Source: BMC Anesthesiol. 2022 Mar 31;22:88. doi: 10.1186/s12871-022-01631-7 (PMC8969381; doi:10.1186/s12871-022-01631-7)
Supplement: Supplementary file 6 — Additional file 6: Supplemental Table 5. Catheter misuse in relation to risk of catheter damage, detailed by type of catheter. Catheter misuse refers to needle movement, the practice of moving the needle in the cannula prior to puncture or during the catheterization attempt (the manufacturer’s use instructions explicitly warn against such maneuvers). Catheter damage refers to abnormalities in catheter structure, macroscopic or microscopic, such as cuts, tears, compressions or loss of material. [file 12871_2022_1631_MOESM6_ESM.docx]

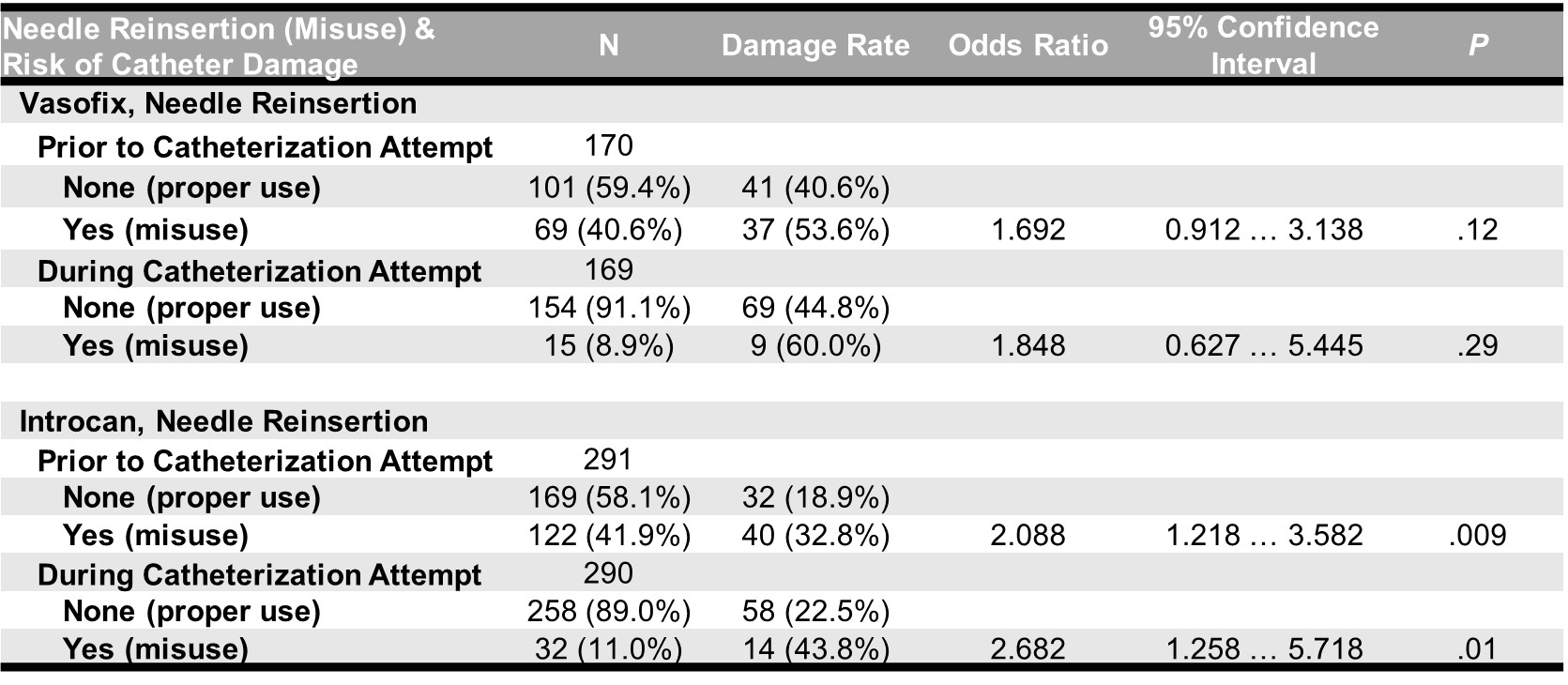


Supplemental Table 5: Catheter misuse in relation to risk of catheter damage, detailed by type of catheter. Catheter misuse refers to needle movement, the practice of moving the needle in the cannula prior to puncture or during the catheterization attempt (the manufacturer’s use instructions explicitly warn against such maneuvers). Catheter damage refers to abnormalities in catheter structure, macroscopic or microscopic, such as cuts, tears, compressions or loss of material.
